# Experimental Investigation of Rheological Properties and Thermal Conductivity of SiO_2_–TiO_2_ Composite Nanofluids Prepared by Atomic Layer Deposition

**DOI:** 10.3390/nano12173014

**Published:** 2022-08-31

**Authors:** Zalán István Várady, Thong Le Ba, Bence Parditka, Zoltán Erdélyi, Klara Hernadi, Gábor Karacs, Gyula Gróf, Imre Miklós Szilágyi

**Affiliations:** 1 Department of Inorganic and Analytical Chemistry, Faculty of Chemical Technology and Biotechnology, Budapest University of Technology and Economics, Muegyetem rakpart 3, 1111 Budapest, Hungary; 2 Department of Solid-State Physics, Faculty of Science and Technology, University of Debrecen, P.O. Box 400, 4002 Debrecen, Hungary; 3 Institute of Physical Metallurgy, Metal Forming and Nanotechnology, University of Miskolc, 3515 Miskolc-Egyetemváros, Hungary; 4 ELKH-ME Materials Science Research Group, ELKH, University of Miskolc, 3515 Miskolc-Egyetemváros, Hungary; 5 Centre of Energy Research, Konkoly-Thege Miklós út 29-33, 1121 Budapest, Hungary

**Keywords:** nanofluid, composite nanoparticles, ALD, viscosity, thermal conductivity, SiO_2_, TiO_2_ layer

## Abstract

In the current research, surface-modified SiO_2_ nanoparticles were used upon immersion in an applied base fluid (ethylene glycol:water = 1:1). The atomic layer deposition method (ALD) was introduced to obtain a thin layer of TiO_2_ to cover the surface of SiO_2_ particles. After the ALD modification, the TiO_2_ content was monitored by energy dispersive X-ray spectroscopy (EDS). Transmission electron microscopy (TEM) and FT-IR spectroscopy were applied for the particle characterization. The nanofluids contained 0.5, 1.0, and 1.5 volume% solid particles and zeta potential measurements were examined in terms of colloid stability. A rotation viscosimeter and thermal conductivity analyzer were used to study the nanofluids’ rheological properties and thermal conductivity. These two parameters were investigated in the temperature range of 20 °C and 60 °C. Based on the results, the thin TiO_2_ coating significant impacted these parameters.

## 1. Introduction

Nanofluid is a phase colloid in which solid particles improve the base fluids’ thermal properties. The used particles are called "nanoparticles" due to their small size (1–500 nm). Base fluids are typically industrial heat transfer fluids such as water, ethanol, ethylene glycol or oils.

In the 19th centuy, Maxwell found a correlation between the dispersed system’s thermal conductivity and its components’ thermal conductivity. Solid materials have much higher thermal conductivity than conventional heat transfer fluids. Almost a hundred years later, in the 1990s, Choi and Eastman increased fluids’ thermal conductivity by adding solid particles and created the term nanofluid [1]. From this point, nanofluids as a field of research gathered significant interest among the scientific community. Taylor et al. compared the volume of published articles to an exponential equation in 2013 [2]. The number of published articles has been growing steadily in recent years.

Nanofluids have opened a new chapter in the history of the thermal properties of fluids. Nowadays, further modification of the particles or the combination of the different particles can allow for obtaining more effective nanofluids. The hybrid and the composite systems mean improved types of nanofluids [3]. Both terms imply that two (or more) kinds of solid materials are present in the base fluid but in different forms. The hybrid nanofluids contain a physical mixture of nanoparticles of two (or more) types; for instance, SiO_2_ and TiO_2_, as in the research of Hamid et al. [4,5,6,7,8,9,10] and Thong et al. [11]. The ZrO_2_–CeO_2_ hybrid nanofluid was investigated by Vidhya et al. [12]. At the same time, the composite nanofluid contains particles where the two phases have chemical bonding, like core–shell particles, where the core and the shell is composed of different materials. The particles’ components are similar to what is used in "simple" nanofluids, such as metals, different oxides or carbon-based materials, and sometimes different polymers attached to the particles. Table 1 shows some examples.

Colloid systems have several different applications. Composite oxide nanoparticles as active components of colloid systems are often used as catalysts. They are useful in wastewater cleaning systems or catalyzing chemical reactions. Wu at al. used CeO_2_-modified Ni-MOF nanoparticles for the electrocatalytic oxidation of urea [25]. Bakos et al. used the method of ALD to prepare TiO_2_ and ZnO single- and multilayer covered carbon nanotubes [26]. The photocatalytic and gas sensing activity of these particles were investigated. 

Some research groups applied an innovative solution to increase the heat capacity of the nanofluids [13,14] using core–shell particles. The core melts at a lower temperature when immersed in the nanofluid and a stable and coherent outer shell protects the melted core. The enthalpy change of phase transition stores the energy (heat). In the two cited examples, Sn particles with SiO_2_ or Al_2_O_3_ covering were used as phase change material, and the base fluids were solar salt (solar salt composes of NaNO_3_ and KNO_3_) and Therminol 66 thermal oil.

There are some articles where composite particles were used to improve the thermal properties of the base fluid. For instance, Arsana et al. investigated the composite SiO_2_–TiO_2_ nanofluids; however, the 640 nm particle size choice is somewhat questionable, affecting the nanofluid stability [17]. Gil-Font et al. used the method of molecular layer deposition to obtain Sn-PET core–shell particles and make stable nanofluids from them with Therminol66 thermal oil as a base fluid to increase the heat transfer coefficient [15]. Shang et al. made Ag-Al_2_O_3_ core–shell particles by atomic layer deposition to increase the optical absorption property of the Therminol66 base fluid [16]. Previously, there were only a few articles about the effect of ALD-modified nanoparticles on the heat transfer properties of a colloid system [21], and the feature of our composite nanofluids has never been investigated.

Our institute has previously studied different nanofluids, such as metal-oxide nanofluids [11], carbon-based nanofluids [27,28], and nanofluids with nanotubular clay particles [29]. In the current research, we used SiO_2_ nanoparticles with a thin TiO_2_ modification on their surface with an average particle diameter of around 20 nm. The TiO_2_ coating was achieved by atomic layer deposition. The particles were investigated using TEM-EDS, and FT-IR techniques. The nanofluids were prepared with these particles in 0.5, 1.0, and 1.5 volume % dispersed in EG: W 1:1 base fluid. The aggregative stability was investigated by following the zeta potential. The thermal conductivity and the rheological properties were also measured.

## 2. Materials and Methods

### 2.1. Materials

SiO_2_ nanoparticles and SiO_2_–TiO_2_ composite nanoparticles were used as solid particles, while ethylene glycol and water served as the components of the base fluid. The SiO_2_ nanopowder (99.5%, 10–20 nm, CAS: 7631-86-9) and ethylene glycol (≥99%, CAS 107-21-1) were purchased from Sigma Aldrich (Budapest, Hungary). Deionized water was produced in the Department of Inorganic and Analytical Chemistry laboratory, Budapest University of Technology and Economics (Budapest, Hungary. The surface-modified composite “SiO_2_–TiO_2_” particles were prepared in a Beneq TFS-200-186 flow type thermal ALD reactor with TiCl_4_ and H_2_O precursors at 108 °C. At this temperature, amorphous TiO_2_ can be expected [30]. Table 2 shows the specific parameters of the atomic layer deposition. The total number of cycles was equal to 410.

### 2.2. Preparation of the Nanofluid

Table 3 presents some properties of the commodities of the nanofluids. The particle diameter of the particles is based on Brunauer-Emmett-Teller theory. The nanoparticle’s density was determined by measuring the weight and the water displacement of a small volume of nanoparticles in a measuring flask. Other parameters were provided by the manufacturer (www.sigmaaldrich.com, 1 July 2022) or from the ASHRAE handbook.

Small volumes (3 mL) of nanofluids were made in 0.5 volume% of composite particles in different EG:W ratios (1:0; 1:1; 1:3; 1:5 and 0:1) to determine which base fluid composition resulted in optimal stability. Higher ethylene glycol content enhances the nanofluid stability, but since water is more environmentally friendly, cheap, and has a lower viscosity, the ideal ethylene glycol: water ratio was selected as 1:1.

This ratio was used to prepare larger amounts (30 mL) of nanofluids in 0.5, 1.0, and 1.5 volume % of composite particles. Nanofluids in the same particle concentrations were also made with the non-modified SiO_2_ particles as a reference. The particles were dispersed by sonication at 130 W and 45 kHz using an ultrasonic bath for 1 h. All the subsequent experiments were performed with these nanofluids. Table 4 contains the summary of the used nanofluids.

### 2.3. Characterisation Methods

Before the nanofluid preparation, the particles were examined. The TEM images and the EDS studies were made by a Philips CM20 Transmission Electron Microscope (Amsterdam, The Netherlands) at 200 kV. The sample preparation was the usual, namely, the particles were dispersed in ethanol, and a small drop from this colloid system was dried onto a copper grid.

The infrared spectra of the particles were studied by Excalibur FTS 3000 BioRad FTIR (Hercules, CA, USA) in the 400–4000 cm^−1^ range in a transmittance mode. The resolution of the measurements was 4 cm^−1^, and the number of accumulated scans was 128. Little samples were mixed with KBr and pressed by a mechanical press to obtain the pastilles for this measurement.

A Brookhaven ZETAPALS (New York, NY, USA) instrument was used for the zeta potential measurement of the different nanofluids. The zeta potential was calculated from the electrophoretic mobility of particles using the Henry equation by considering the Smoluchowski approximation. Three replicates of each sample were tested, and an average value was reported.

An Anton Paar Physica MCR 301 (Ashland, VA, USA) rotation viscosimeter was used to characterize the rheological behavior of the SiO_2_ and the composite nanofluids at different shear rates and five different temperatures of 20, 30, 40, 50, and 60 °C. The number of data points per measurement was equal to 15. The amplitude was 5%, and the angular frequency range was 0.6 to 3600 s^−1^.

The thermal conductivity of SiO_2_ and the composite samples was measured based on the modified transient plane source technique using an SKZ1061C TPS thermal conductivity analyzer (Jinan, Shandong, China). The thermal conductivity of all samples was measured at five different temperatures of 20, 30, 40, 50, and 60 °C. Three thermal conductivity measurements were performed for each sample, and an average value was reported.

## 3. Results and Discussion

### 3.1. SiO_2_ and the Composite Particles

**Figure 1:**
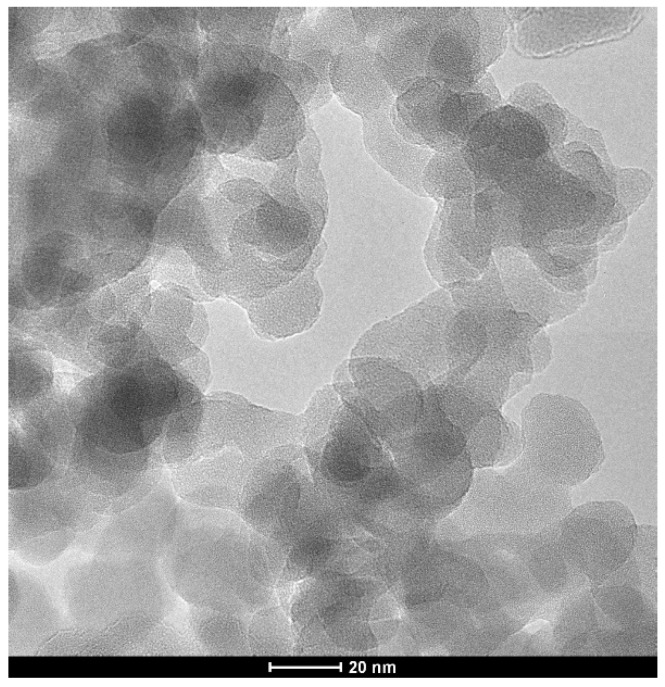
TEM images of the SiO_2_ particles.

Figure 1 shows the TEM images of the SiO_2_ particles. The spotty texture of the particles indicates the amorphous phase. However, the particles may seem a bit larger than 10-20 nm, as the data in Table 2 are from the BET method, which means our particles have the same specific area as the regular spheres of 10–20 nm.

**Figure 2:**
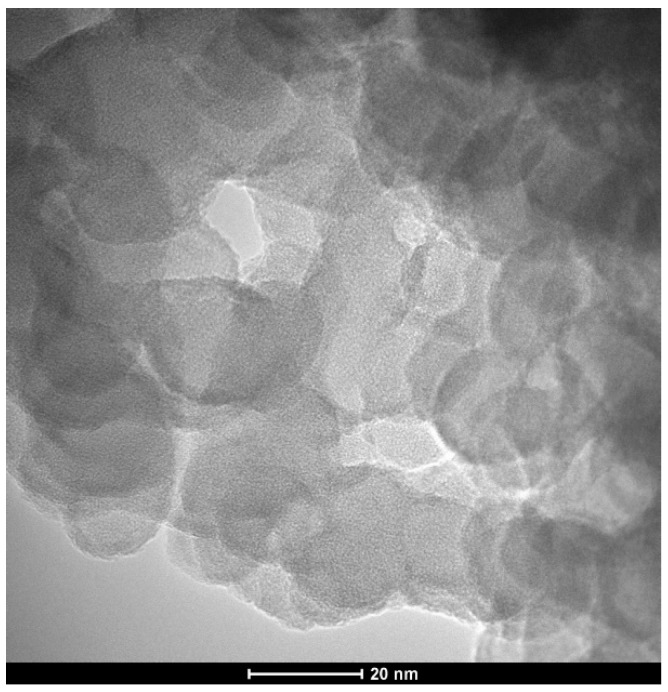
TEM micrograph of the composite particles.

Figure 2 provides a TEM micrograph of composite particles. The darker outline is caused by the appearance of atoms with larger electron clouds on the surface of these particles.

**Figure 3:**
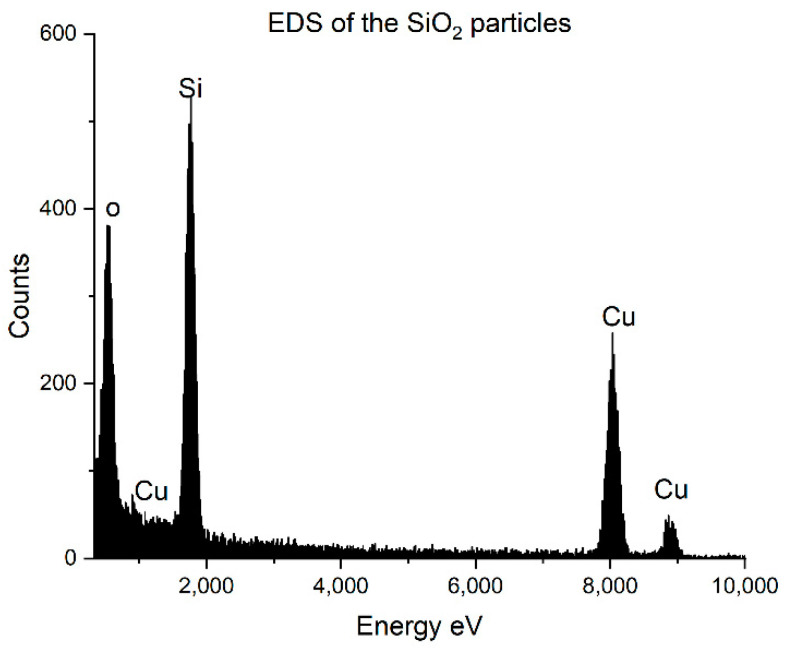
EDS of the SiO_2_ particles.

Energy-dispersive X-ray spectroscopy measurements were made to further prove the success of the atomic layer deposition reaction. In Figure 3, the EDS spectrum of the SiO_2_ particles is seen. The Si and O atoms come from the particles, and copper (Cu) is the material of the grid.

**Figure 4:**
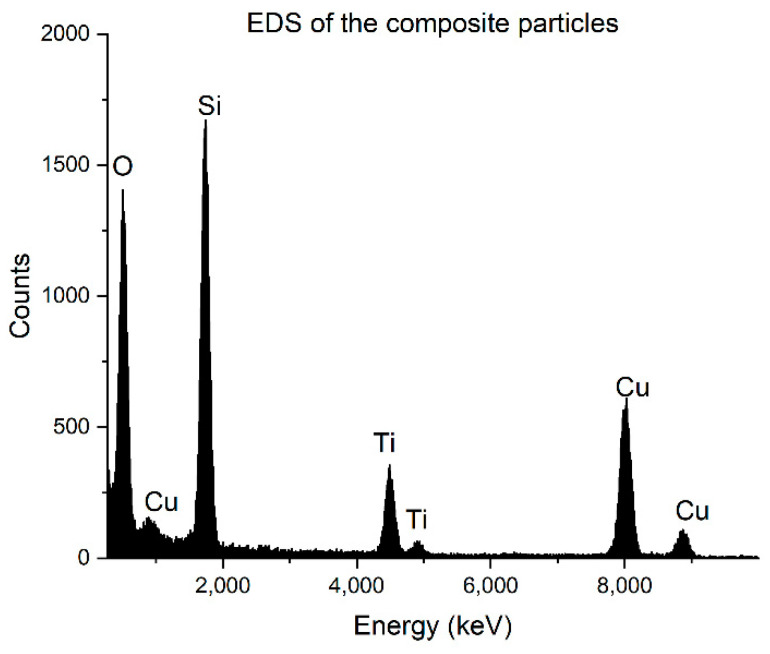
EDS of the composite particles.

In Figure 4, the EDS spectrum is seen from the composite particles. The characteristic peaks of Ti are clearly seen in this spectrum; thus, the darker cover must have been TiO_2_.

**Figure 5:**
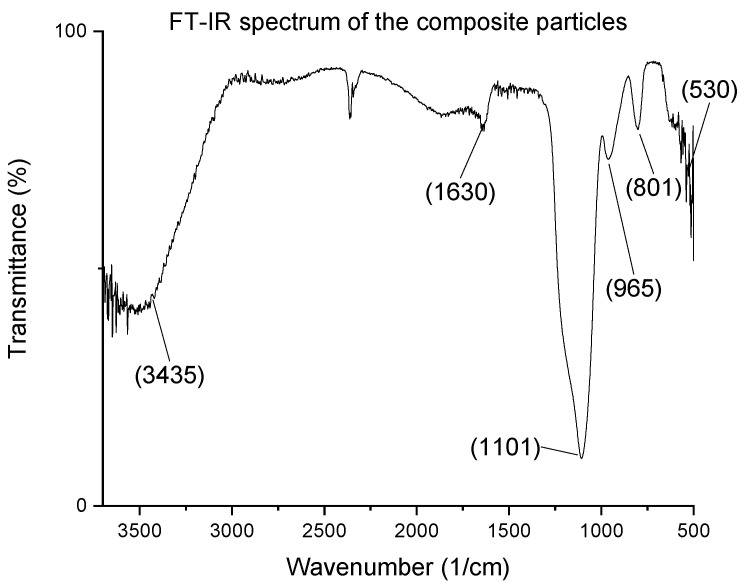
FT-IR spectrum of the composite particles.

Fourier transform infrared spectra (FT-IR) was also performed, but the TiO_2_ coating was undetectable by this method. Thus, only the composite particles’ IR spectrum is demonstrated in Figure 5. Table 5 shows the identified IR peaks characteristic of the amorphous SiO_2_ [31], and the CO_2_ content of the air caused a small peak at around 2300 cm^−1^.

### 3.2. Zeta Potential Measurements

The aggregative stability of the different nanofluids was characterized by the zeta potential, as demonstrated in Table 6.

The nanofluid is considered stable if the zeta potential value is outside the range ±30 mV. The composite nanofluids are stable; however, the pure SiO_2_ is even more stable since the zeta potential values are generally greater. For the pure SiO_2_ nanofluids, the larger zeta potential is caused by the increasing volume% of SiO_2_ particles due to a higher negative electrical charge.

According to the visual observations, all nanofluids were stable at room temperature for more than a week.

### 3.3. Rheological Properties

**Figure 6:**
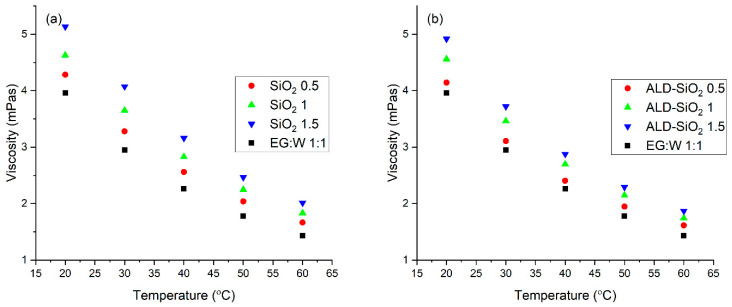
The viscosity of the SiO_2_ (**a**) and composite nanofluids (**b**).

The rheological properties of these nanofluids can be considered Newtonian with a good approximation, meaning that viscosity can be considered as the characteristic parameter. Figure 6 presents the viscosity of the pure SiO_2_ nanofluids and the composite nanofluids at five different temperatures between 20 °C and 60 °C.

The viscosity of all nanofluids decreased with the increase of the temperature, similarly to the base fluid. Not surprisingly, the increasing volume fraction of the nanoparticles increases the viscosity of the nanofluid, similarly to most nanofluids in general.

**Figure 7:**
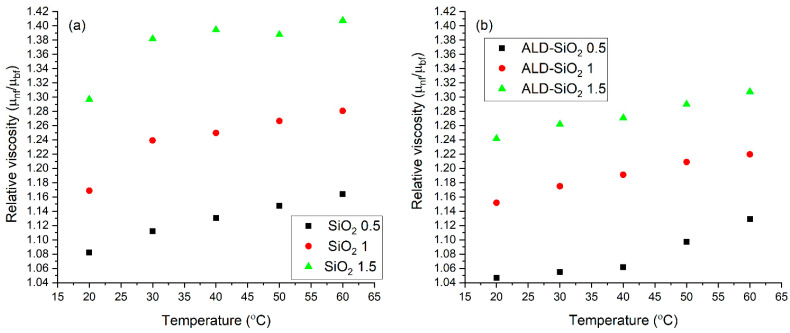
The relative viscosity of the SiO_2_ (**a**) and composite nanofluids (**b**) as a function of temperature.

The graphs depicted in Figure 7 demonstrate the relative viscosity of pure and composite nanofluids over the viscosity of the base nanofluid (ethylene glycol: water= 1:1). In the case of the composite nanofluids, surface modification decreases the relative viscosity by approximately 5%. This is an interesting result, meaning that the very thin ALD coating is beneficial in reducing the viscosity here.

### 3.4. Thermal Conductivity

**Figure 8:**
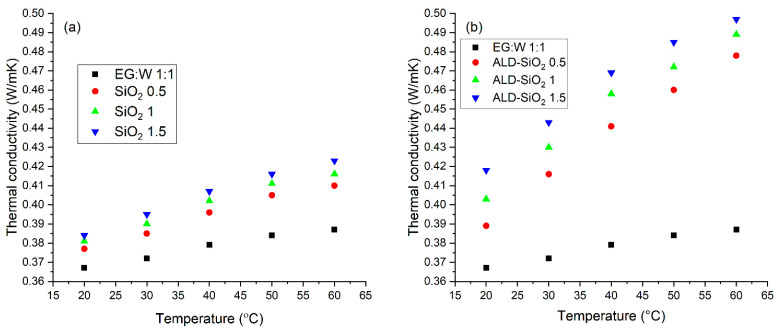
Thermal conductivity of the SiO_2_ (**a**) and the composite (**b**) nanofluids.

Figure 8 shows the thermal conductivity of the nanofluids. The increasing volume fraction of the particles increases the thermal conductivity, but the surface modification has an even more dramatic effect on increasing the thermal conductivity.

**Figure 9:**
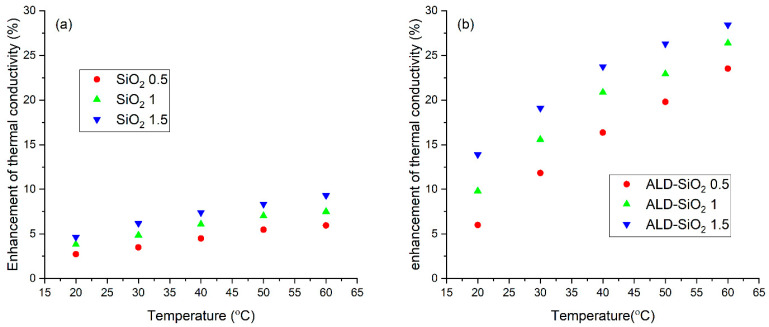
Enhancement of thermal conductivity of the SiO_2_ (**a**) and composite (**b**) nanofluids.

Figure 9 presents the rise of the thermal conductivity compared to the base fluid. An increase of only 2–10% was observed using the pure SiO_2_ particles, but the composite particles caused a more significant (5–28%) change. It means that the composite particles affect 3.2 times better thermal conductivity than the SiO_2_ particles do.

In our previous work, when SiO_2_-P25 TiO_2_ hybrid nanofluids were used, the maximal increase was only 12% when 1.5 vol% nanoparticles were used, and the temperature of the thermal conductivity measurement was 60 °C [11]. Though the ethylene glycol to water ratio was different, the effect of the pure SiO_2_ particles was similar in terms of an increase in thermal conductivity. Thus, the mixing the different particles can be beneficial to obtain a better nanofluid, but sometimes the further modification of the particles can cause a larger increase in thermal conductivity.

### 3.5. Regression

**Figure 10:**
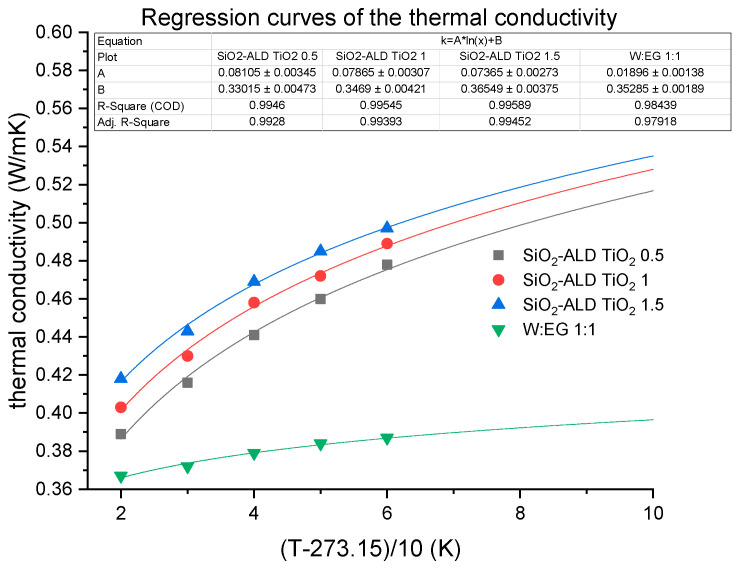
Regression curves of the thermal conductivity.

According to our measurement data, the SiO_2_–TiO_2_ composite nanofluid made on the 1:1 deionized water–glycol base fluid, the hermal conductivity can be calculated as follows:

$$\left(k=A\cdot \ln\left(\frac{T-273}{10}\right)+B\right)$$

where A and B terms are according to the small table in Figure 10 and T should be substituted in Kelvin.

Since the regression’s *R*^2^ values are high, the extension above the measurement range can be applied up to 373 K (100 °C).

### 3.6. Balancing of the Viscosity and Thermal Conductivity

An intensification of the heat transfer processes means decreasing the temperature range that exists during the heat transfer process. According to the second law of thermodynamics, the reduced temperature change means increased generation of entropy. This greater entropy generation in heat transfer processes appears as the increased technical work, i.e., pumping work. In other words, the second law of thermodynamics states that it is impossible to intensify the heat transfer without encouraging the generation of entropy. In technical processes, the transferred heat equals the change of enthalpy and the technical work done during the process. When the heat transfer is intensified, fewer temperature change occur, which means the change in enthalpy is smaller. Maintaining the same heat transfer, the technical work—pumping work—increase occurs according to the first law of thermodynamics.

The Nusselt number characterizes the heat transfer intensity, while the technical work needed for maintaining the process depends on the Reynolds number. The higher thermal conductivity in the case of the same Nusselt number means a higher heat transfer coefficient. The higher viscosity results in decreased Reynolds number in case of the same fluid velocity. Since the Nusselt number is a monotonous function of the Reynolds number, the decreased Reynolds number results in a decreased Nusselt number. Maintaining the Reynolds number to its original value needs increased velocity, which means increased pumping work. These phenomena can be the “scientific base” for balancing thermal conductivity and viscosity. The balancing itself is an engineering issue since there is no general solution. Every system needs identical analyses. The heat transfer systems should meet several requirements, and the cost of operation is a significant point. The pumping work is a major factor in the operation cost. In that context, it is an engineering design task to determine the proper operation parameters, including the optimal working fluid and its properties.

### 3.7. Future Research Directions

The modification of the surface can cause different changes. For instance, it can eliminate surface irregularities, thus making the particles more suitable for heat transfer, or it can increase the amount of -OH groups on the surface, which can cause more vital interaction between the surface and the fluid. It can change the pH on the surface, or it can make the particles more dispersible.

In future work, we would like to investigate other composites (ALD modified) oxide nanofluids to find regularity between the changes and understand the effects more clearly to know to build more suitable particles for heat transfer applications.

## 4. Conclusions

Pure SiO_2_ and composite ALD TiO_2_–SiO_2_ particles were used to make nanofluids in three different concentrations (0.5, 1.0 and 1.5 vol%). The base fluid was EG:W = 1:1. All of the particles caused a rise in the viscosity and the thermal conductivity values. The simple particles caused 8–41%, but the composite particles caused only a 4–31% viscosity increase, as represented in Figure 5. Therefore, the increase of viscosity can be moderated by surface modification. In terms of thermal conductivity, the pure SiO_2_ particles caused only a 2–10% increase, whereas the modified particles caused a rise between 5–28%, so by the introduction of TiO_2,_ both parameters can be favorably influenced.

Although the ethylene glycol–water ratio was different when the separated SiO_2_ and TiO_2_ particles were used, in one of our previous studies [11], the SiO_2_ particles were used from the same batch so the changes in the effects might be comparable. When the hybrid nanofluid was used, the thermal conductivity was increased by 1.71 times than the increase observed using only the SiO_2_ particles. In this present study, the composite particles caused a 3.22 times greater change on average than the SiO_2_ particles. Using the SiO_2_ and TiO_2_ particles, the viscosity increase was, on average, 1.30-fold greater than using only SiO_2_ particles. However, in this study, the viscosity increase affected by the particles was 1.46 times smaller when the composite particles were used instead of the simple ones.

Modifying the particles’ surfaces can effectively create more specialized nanofluids. ALD is an up-and-coming method in nanofluid research. As such, it would be necessary to investigate such changes, but it may be assumed that the ALD caused the morphological and chemical changes in the surface of the particles, resulting in varied flow conditions.

## Figures and Tables

**Table 1:** Examples of composite particles used in nanofluids.

Authors	Material of the Base	Coating Material	Coating Method
Cingarapu et al. [13]	Sn	SiO_2_	sol–gel silica encapsulation process
Navarrete et al. [14]	Sn	SiO_2_ or Al_2_O_3_	ALD
Gil-Font et al. [15]	Sn	Polyethylene terepthalate	Molecular layer deposition
Shang et al. [16]	Ag	Al_2_O_3_	ALD
Arsana et al. [17]	SiO_2_	TiO_2_	-
Botha et al. [18]	SiO_2_	Ag	Chemical reaction
Bhanvase et al. [19]	CuO	polyaniline	In situ emulsion polymerization
Chakraborty [20]	Cu-Al layered double hydroxides		One pot chemical reaction
Bohus et al. [21]	Carbon nanosphere or carbon nanopowder	TiO_2_	ALD
Mehrali et al. [22]	Graphene oxide nanosheets	Ag	Chemical reaction
Sundar et al. [23]	MWCNT (multiwall carbon nanotube)	Fe_3_O_4_	In situ chemical reaction.
Sundar et al. [24]	C (nanodiamond)	Fe_3_O_4_	Chemical reduction

**Table 2:** Parameters of the atomic layer deposition.

Chamber pressure, mbar	6.3
Reactor pressure, mbar	1.3
TiCl_4_ pulse time, ms	300
H_2_O pulse time, ms	300
TiCl_4_ purge time, ms	3000
H_2_O purge time, ms	3000
Temperature, °C	108
Number of cycles	410

**Table 3:** Properties of the applied particles and fluids.

Properties	SiO_2_ Particles	Composite Particles	Ethylene Glycol	Water
color	white	white	limpid	limpid
Molecular mass, g/mol	60.08	-	62.07	18.02
Average particle diameter, nm	10–20	11–21	-	-
Density, at 20 °C, kg/m^3^	2138 ± 50	2150 ± 50	1113	997
Melting point, °C	2230	-	−12.7	0
Boiling point, at 101.3, kPa	-	-	198	100
Viscosity, at 20 °C, mPas	-	-	20.9	1.00
Thermal conductivity, W/mK	-	-	0.258	0.609
Specific heat, at 20 °C, J/kgK	-	-	2347	4186

**Table 4:** Composition of the SiO_2_ and the composite nanofluids.

Sample Name	Nanoparticle vol%	Base Fluid vol%
SiO_2_-ALD TiO_2_ 0.5	0.5	99.5
SiO_2_-ALD TiO_2_ 1.0	1.0	99.0
SiO_2_-ALD TiO_2_ 1.5	1.5	98.5
SiO_2_ 0.5	0.5	99.5
SiO_2_ 1.0	1.0	99.0
SiO_2_ 1.5	1.5	98.5

**Table 5:** Vibrations of the composite nanoparticles.

Wavenumber (cm^−1^)	Vibration
3435	O-H stretching (from water)
3246 (appears in the shoulder)	Si-OH stretching
1630	H-O-H bending (from water)
1384	Si-O stretching
1101	O-Si-O asymmetrical stretching
961	Si-OH
801	O-Si-O symmetrical stretching
475	Si-O-Si stretching

**Table 6:** Average zeta potential values of the different nanofluids.

Sample Name	Zeta Potential (mV)
SiO_2_-ALD TiO_2_ 0.5	−30.25
SiO_2_-ALD TiO_2_ 1.0	−33.62
SiO_2_-ALD TiO_2_ 1.5	−32.86
SiO_2_ 0.5	−32,82
SiO_2_ 1.0	−33.03
SiO_2_ 1.5	−44.85

## Data Availability

The data presented in this study are available on request from the corresponding authors.
